# Local Knowledge and Conservation of Seagrasses in the Tamil Nadu State of India

**DOI:** 10.1186/1746-4269-7-37

**Published:** 2011-11-23

**Authors:** AF Newmaster, KJ Berg, S Ragupathy, M Palanisamy, K Sambandan, SG Newmaster

**Affiliations:** 1 Biodiversity Institute of Ontario Herbarium, University of Guelph, Guelph, Ontario N1G 2W1, Canada; 2 Botanical survey of India, Southern Regional Centre, Coimbatore-641003 Tamil Nadu, India; 3 Post Graduate Department of Plant Science, Avvaiyar Government College for Women, Karaikal 609 602, U.T. of Puducherry, India

## Abstract

Local knowledge systems are not considered in the conservation of fragile seagrass marine ecosystems. In fact, little is known about the utility of seagrasses in local coastal communities. This is intriguing given that some local communities rely on seagrasses to sustain their livelihoods and have relocated their villages to areas with a rich diversity and abundance of seagrasses. The purpose of this study is to assist in conservation efforts regarding seagrasses through identifying Traditional Ecological Knowledge (TEK) from local knowledge systems of seagrasses from 40 coastal communities along the eastern coast of India. We explore the assemblage of scientific and local traditional knowledge concerning the 1. classification of seagrasses (comparing scientific and traditional classification systems), 2. utility of seagrasses, 3. Traditional Ecological Knowledge (TEK) of seagrasses, and 4. current conservation efforts for seagrass ecosystems. Our results indicate that local knowledge systems consist of a complex classification of seagrass diversity that considers the role of seagrasses in the marine ecosystem. This fine-scaled ethno-classification gives rise to five times the number of taxa (10 species = 50 local ethnotaxa), each with a unique role in the ecosystem and utility within coastal communities, including the use of seagrasses for medicine (e.g., treatment of heart conditions, seasickness, etc.), food (nutritious seeds), fertilizer (nutrient rich biomass) and livestock feed (goats and sheep). Local communities are concerned about the loss of seagrass diversity and have considerable local knowledge that is valuable for conservation and restoration plans. This study serves as a case study example of the depth and breadth of local knowledge systems for a particular ecosystem that is in peril.

Key words: local health and nutrition, traditional ecological knowledge (TEK), conservation and natural resources management, consensus, ethnomedicine, ethnotaxa, cultural heritage

## Introduction

Seagrasses are an artificial grouping of grass-like plants that grow in or around aquatic marine ecosystems. The name seagrass is purely descriptive as is the name seaweed with respect to marine algae [1]. Despite popular misunderstanding of the taxonomic relation of seagrasses to the grass family (Poaceae), seagrasses in fact have no relation to grasses as supported by systematic and phylogenetic evidence. However, the term seagrass aptly defines a group of angiosperms that are specially adapted to grow in estuaries and marine ecosystems. As such, this group of plants includes 13 genera and approximately 72 species that belong to the families Zosteraceae, Potamogetonaceae, Posidoniaceae, Cymodoceaceae, Hydrocharitaceae and Ruppiaceae [2,3].

Seagrasses have adapted to grow in coastal marine environments in both tropical and temperate regions on almost every continent in the world [2,4]. In tropical seas, genera such as *Cymodocea, Enhalus, Halodule, Halophila, Syringodium, Thalassia *and *Thalassodendron *are represented. However, some species of these genera are also found in temperate regions, whereas species of *Amphibolis, Heterozostera, Phyllospadix, Posidonia, Pseudalthenia *and *Zostera *are usually restricted only to temperate seas [5-7]. Growing erect from rhizomes embedded in the sediment and debris on the ocean floor, seagrasses are the only angiosperms that are able to thrive underwater in marine environments [2]. In general seagrasses inhabit the tidal and subtidal zones of shallow and sheltered localities of seas, gulfs, bays, backwaters, lagoons and estuaries. They usually prefer muddy, sandy, clayey and coral rubble substrate, but they also grow on rocks and in crevices. They are found to grow either homogenously or heterogeneously, forming thick and dense meadows that produce considerable biomass, provide excellent habitat, and perform multiple ecosystem services for some of the world's most biodiverse and productive marine ecosystems [8]. Some of the important ecosystem services attributed to seagrasses include the recycling of nutrients, and the stabilization of seafloor sediment, which prevents erosion during storms and provides critical habitat for spawning fish and many juvenile marine invertebrates. Seagrasses also serve as habitat and a food source. They serve as the primary food for green sea turtles, manatees and dugongs [2,4], and they support a rich variety of fish, which in turn attracts a diversity of predators including a rich diversity of birds and some large mammals.

The importance of seagrasses has been documented in many coastal communities around the world, including India [9], Africa [10], Canada [11,12], Mexico [13,14] and Sweden [15]. Some of these studies describe the role of seagrasses in ecosystem function [15], while others highlight their economic and traditional value, such as the research of Wyllie-Echeverria and Cox [11] concerning wild and commercial gathering of seagrass (*Zostera marina*) by North American fishing communities during the early to mid 1900s for use as green manure and insulating products. Seagrasses have also been studied for their potential use in modern medicine [9,16-18]. Recent research on seagrass phyochemistry has shown that they are an important source of antioxidants [9], antibacterial agents [17], minerals [18] and possibly anticancer compounds [19].

The literature documenting the traditional knowledge of seagrasses is sparse, but there are a number of important sources that describe the plant's unique value and varied uses within coastal indigenous societies. Communities that value seagrass are often suitably located in coastal areas of abundant seagrass meadows that serve as rich and accessible habitat for fishing, and from which seagrass can be easily gathered for use as garden fertilizers [20] and as a source of food [21]. In India, coastal indigenous people claim that their ancestors have used seagrasses for thousands of years for a variety of uses from food to medicine [22,23]. Similarly, seagrass (the eelgrass *Z. marina*) was a vitally important source of food and medicine, and had both cultural and socio-economic value for the Seri Indians from the Gulf of California in Sonora, Mexico. Studies by Felger and Moser [13,14] reveal that not only did eelgrass supply the Seri with their key grain resource, but eelgrass was also a primary food of green sea turtles (*Chelonia*), the single most important traditional food of the Seri. The authors describe how, as a reflection of the central role of eelgrass in Seri culture, eelgrass is prominently imprinted in the Seri language, and Seri elders retain detailed knowledge of the distribution and ecology of eelgrass within their territory. The eelgrass *Z. marina *was also favored by several coastal groups of British Columbia, but instead of harvesting the seeds, the eelgrass was gathered for consumption of its rhizomes and leaf bases, or for collecting herring eggs in the spring [12].

In addition to research by Felger and Moser [14], there is only one other published report [21], to our knowledge, on the medicinal use of seagrasses by an indigenous community. This study explores the interactions between seagrasses and coastal dwelling stakeholders in Chwaka Bay, on the East Coast of Zanzibar, Africa. However, rather than working from surveys or quantitative analyses, the research is based only on qualitative data, simply listing seagrasses and their associated use for ethnomedicine. It is our view that rigorous experimental design and consensus analysis of traditional knowledge among informants provides quantitative support for local knowledge that may impact society-at-large [24,25]. There is an immediate need to quantitatively survey the traditional knowledge of seagrasses in areas where they are abundant and serve as an important resource to coastal communities.

Society is in a quandary concerning the conservation of seagrasses [26]. Seagrass ecosystems are being destroyed at a rapid rate [20] in spite of the fact that they support high biodiversity and sustain many cultures as a vital source of their livelihood [27,28]. Conservation measures for protecting these biodiversity hotspots have been established in North America and Australia, and countries such as India have been included in the Coastal Regulation Zone Act to protect species such as seagrasses [8]. However, seagrasses are rarely considered in resource management, education and ecological conservation plans. In order to conserve and raise awareness on these vital plants, political mandates need to be informed by the perspective of traditional knowledge and the associated value to coastal communities around the world that reside near seagrass ecosystems.

India has a vast coastal belt extending up to 6000 km, which provides excellent habitat for seagrasses. This coastal area is immense (2,013,440 sq. km) and has conflicting land resource management needs; on one hand, there is rapid, ongoing development of India's Exclusive Economic Zone (EEZ); the EEZ is a zone that extends for a distance of 200 nautical miles seaward from the coastal edge of a nation's territorial waters. Within this zone, a nation has rights to exploit marine resources, primarily in the form of fishing and seabed mining, but also including the harvesting of energy from water and wind [29].

There is also an urgent need for biodiversity conservation within a large (452,060 sq. km), unique coastal oceanic shelf [29]. This shelf includes the Bay of Bengal, spanning from West Bengal to Kodikarai Tamil Nadu, and includes estuaries fed by five major rivers in Tamil Nadu, namely Palar, south Pennar, Kollidam, Cauvery and Thamiraparani. These rivers all play a major role in the growth of seagrass along the river mouths of the estuaries. This is an inimitable biosphere inhabited by a diversity of cultures that have utilized resources in a sustainable way for many years, yet it lacks protection [30]. The Coromandel Coast comprises a major part of the Bay of Bengal, the Palk Bay and the Gulf of Mannar, and a wide variety of unique landscapes along this coastline contributes to a rich diversity of plant communities; seagrass habitats within this zone are supported by sheltered bays, gulfs, straits, channels, lagoons, estuaries, salt water lakes and backwater ponds. Thirteen seagrass species are found in the Tamil Nadu state, including: *Enhalus acoroides *(L.F.) Royle*, Halophila beccarii *Asch., *Halophila decipiens *Ostenf*., Halophila stipulacea, Halophila ovalis *(R.Br.) Hook. F, *Halophila ovalis ssp. ramamurthiana, Halophila ovata *Gaud*., Thalassia hemprichii *(Ehrenb.) Asch*., Cymodocea rotundata *Ehrenb. & Hempr. Ex Asch*., Cymodocea serrulata*, (R.Br.) Asch. & Magnus*, Halodule pinifolia *(Miki) Hartog*, Halodule uninervis *(Forssk.) Asch*.*, and *Syringodium isoetifolium *(Asch.) Dandy [20].

In India, detailed distributional or taxonomical information on seagrasses is sparse, and ethnobotanical literature is lacking. There are a number of studies in which seagrasses were included in floristic surveys in India, and across these studies, a total of only 12 seagrass species were reported [20,31-34]. However, there is only limited research that exclusively focuses on seagrasses in a few locations [20]. We are not aware of any publication on the traditional or local knowledge of seagrasses in India. The purpose of this study is to assemble the local Traditional Ecological Knowledge (TEK) of seagrasses from communities along the eastern coast of India in order to assist in conservation of marine ecosystems. More specifically, we explore scientific and traditional local knowledge concerning the 1. classification of seagrasses (comparing scientific and traditional classification systems), 2. utility of seagrasses, 3. Traditional Ecological Knowledge (TEK) of seagrasses, and 4. current conservation efforts for seagrass ecosystems.

## Methods

### Study site

**Figure 1:**
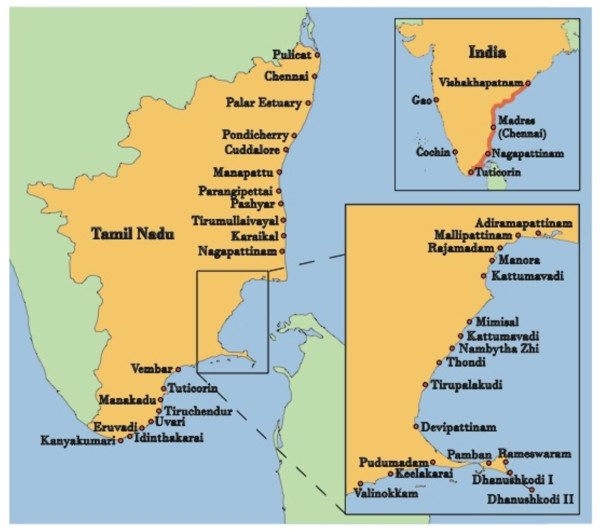
**Study area including 40 villages along the eastern coast of India from Chennai (Pulicat-lake) to Kannyakumari in the state of Tamil Nadu**.

The area of study included the eastern coast of India from Chennai (Pulicat-lake) to Kannyakumari in the state of Tamil Nadu. Sampling for traditional knowledge of seagrasses occurred within 40 rural fishing villages (Figure 1).

### Ethnography

There was exceptional cultural diversity in our study where the Indian Ocean and the Arabian Sea meet (Figure 1), and each of the 40 villages surveyed contained a number of different religious groups, including Hindu, Islam and Christian. Mixed tribal groups who represent the Dravidians, the ancestors of ancient southeastern India, were also represented in the villages [35]. Religions of the tribal groups include Hindu and Christianity, and they speak a number of different dialects that are slightly different from the modern Dravidian [36]. Many people within these groups also speak Islam and Tamil.

### Ethnobotanical Surveys

**Figure 2:**
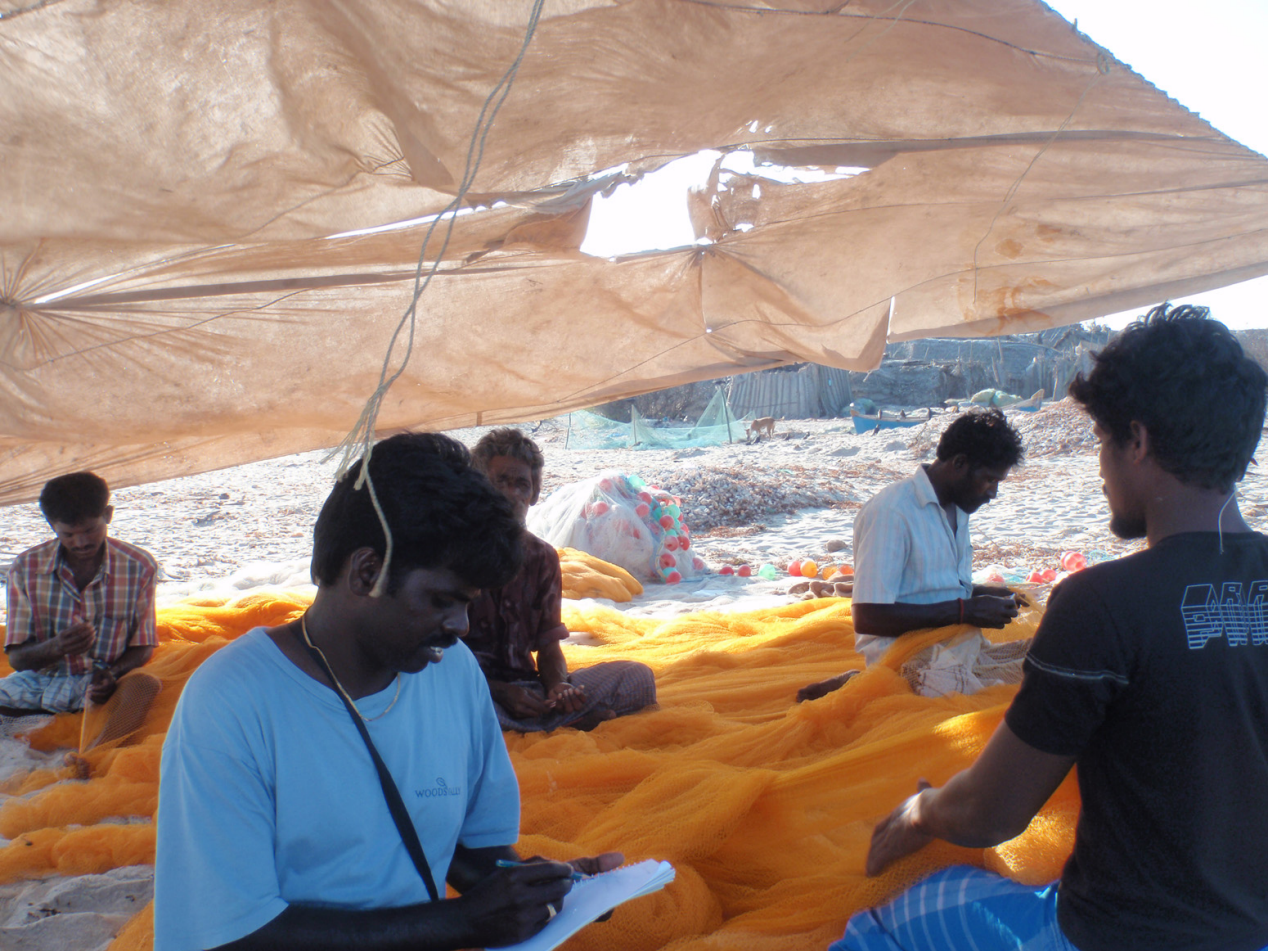
**Interview with a local elder about seagrass traditional knowledge**.

Ethnobotanical explorations were made in the study area in 2011 (Figure 1). Our research team included local "informants" (Figure 2) from the villages, and botanists from the Centre for Biodiversity Genomics (CBG) at the University of Guelph and the Botanical Survey of India. The informants were selected following standard interview protocols [37,38], and key informants of TEK (i.e., local experts, of which two were chosen for each village) were identified using Bernard's [39] methodology. From a total of 130 possible participants we selected 90 informants, including 60 men, 10 women, and 10 of each male and female children. Ages of informants ranged from 10 to 85, and occupations of informants (excluding fishing, which was the most common occupation) included sales, truck driving, tailoring, teaching, local politics, and business. We intended to interview a balanced ratio of men and women, but circumstances prevented us from doing so; the majority of women denied their knowledge of seagrass and refused to be interviewed. Women are directly involved in the processing of marine resources and it may be entirely possible that they have a specialized knowledge of seagrasses, but during our surveys they seemed to spend most of their time working with the fish. It may be for this reason that most women denied to be interviewed. Verbal consent was obtained from all individual participants involved in our surveys. The consent statement presented to participants included the following: If you agree to participate in this study, we will request your involvement each day for 3 days between 8 a.m. to 12 noon. In the case that we need data validation you will be contacted again for an hour if necessary. The interviews will be conducted in your native language, and all interview and consent records will be stored under lock and key. The results of this study will first be published in your language as research notes and distributed to your community, and later, following your consent, the results will be published in a scientific journal.

The field data were collected in a series of unstructured, semi-structured and structured interviews [40,41] over the course of two stages. First, in an initial period of participant observation, researchers accompanied local informants in their boat-based activities and other interactions with seagrasses. For each seagrass species encountered, the plant was identified, a voucher specimen collected and labeled, and informants were surveyed in an unstructured and semi-structured format as to the plant's utility. Second, the collected voucher samples were then the subject of semi-structured and structured interviews in the 40 villages. Using various research protocols such as free recall lists, pile sorts, and consensus analysis [37,38], this stage served to garner additional TEK, validate the identification of voucher samples, and determine differences in knowledge and taxonomy among local informants in other villages. Other interview protocols and methods of data collection and confirmation were based on Pelto and Pelto [42], Etkin [43], Bernard [39], Vogl et al. [44], and Stepp and Thomas [45].

Semi-structured interviews involved flexible, open-ended questions and conversations with informants in fishing boats, at village areas where fishing nets and boats were being prepared, in fish sorting locations, and in both small and large fish markets. At times, visual cues (e.g., photos, plants) were used to trigger memories or insight for informants that may not arise in unfamiliar interview locations (e.g., 46). This technique helps to promote spaces where participants can guide the inquiry process as to the perceptions and values related to seagrasses [47].

Throughout the semi-structured and structured interview process, a number of participatory tools were used to elicit additional traditional uses, identifications, nomenclature, classifications and other associated TEK of each voucher specimen. These tools included Community Biodiversity Registers and Diversity Fairs, a participatory approach that involves exploring ethnotaxa diversity through pile-sorting activities based on names, utility, or other factors [48,49]. Boat-hold surveys were also used to understand (a) the contribution of seagrasses to a family's household activities (i.e., economy, food, medicine) and (b) the access to and control over coastal areas and seagrass resources needed for nutritional self-sufficiency.

Selected key interview questions (using the 'Kadal passi' ethnotaxa as an example):

1) Do you know about 'Kadal passi' seagrass?

2) Do you know this name?

3) Do you ever use this plant?

4) How important is 'Kadal passi' seagrass to you?

5) Do you sell 'Kadal passi' seagrass?

6) If yes, what part of the plant do you sell?

7) Why do you sell this plant?

8) Where do you sell the plant?

9) How often do you sell the plant?

10) How much money do you earn for selling this plant?

11) Is the money you earn enough for your daily meals?

13) What is 'Kadal passi' seagrass used for?

14) From whom did you learn the uses of 'Kadal passi' seagrass?

15) How is the seagrass habitat important to you?

16) How is this ecosystem important to you?

17) What is good about being at this site?

18) What is the name of this area?

19) Why is this important to you?

In addition to members of the fishing community, we also interviewed other actors (e.g. biodiversity resource managers, conservation practitioners) that were relevant to understanding the processes (and discourses) that shaped resource access. For all actors, we conducted oral history interviews to elicit the memories and personal commentaries relevant to coastal activities and resource assess. All interviews were conducted using the Tamil language.

Plant samples of each ethnotaxa were collected from all of the local indigenous communities and preserved for both herbaria and DNA barcode analysis. Leaf, stem and flower parts collected in situ were fixed in silica gel, FAA (50% ethanol, 5% acetic acid, 10% formalin, 35% water) and stored in 70% ethanol for morphological study ex situ. These samples were used for measuring the variation in morphological characters and molecular markers. Herbarium voucher specimens were deposited in the Center for Bio-Cultural Diversity (CBD) in Chennai, India. Scanned herbarium images are available in the OAC Herbarium, Biodiversity Institute of Ontario, University of Guelph, Ontario.

### Identification and TEK Consensus Analysis

Calculation of pile sorting relative frequency (RF) and a consensus factor (FIC) was used to test homogeneity of knowledge (scientific knowledge, SK, and traditional knowledge, TEK) in the identification of specimens, revealing cryptic taxa (ethnotaxa) or limitations of the classification without the use of molecular data. Voucher samples collected from the 40 villages were systematically identified by taxonomists at the University of Guelph Herbarium, the Botanical Survey of India and by local informants at the time of their collection in India. The relative frequency (RF) of each specimen from the interviews was calculated to determine a quantitative value for choosing a plant name (Latin binomial or TEK ethnotaxa) from the pool of collected vouchers placed in a species concept [50]. RF is the simple calculation of the percentage of specimens associated with a taxon when taxonomists or local informants are presented with a pool of vouchers and asked to perform "pile sorting" [37]. Trotter and Logan [51,52] provide the calculation of a consensus factor [Fic=Nur-Nt/(Nur-1)], which was adopted to evaluate the degree of partition into categories [24]. We have adopted this calculation as a means to examine plant utility by the indigenous informants [24,25,53], where Nur is the number of use-reports of informants for a particular category (TEK plant use), where a use-report is a single record for use of a plant mentioned by an individual, and where Nt refers to the number of species used for that particular category for all informants [53,54].

### Seagrass Multivariate Classification Analyses

Bray-Curtis average linkage was used to classify 155 specimens of seagrass ethnotaxa using four TEK classification characters (morphology, ecology, experience, gestalt). These four characters were recorded for all ethnotaxa during the interviews and represent the primary characteristics used by the informants; their use in this study follows methods previously established [50]. Morphological characters included visual morphological features (e.g., leaf size or color). Ecological characters included those associated with sites where the informants collect seagrasses. Experience refers to personal skills used by the informants to identify seagrass ethnotaxa. These experiences are comprised of olfaction (smell), palpation (touch), gustation (taste), and visual and auditory cues. Previous research [50] has demonstrated that some cultures can recognize plants without the use of any specific characters. This is often called "Gestalt" identification; we know some things by the whole and not by its finer physical properties. We recorded the seagrass ethnotaxa that informants could distinguish without reference to any other recognition characters. This classification included plant "utility", which is defined as an informant's use (e.g., nutritional, medicinal, technical or ritual) of seagrass. The similarity matrix of species by characters was used to build a cluster diagram, which is useful in assessing the ability of the characters in classifying all ethnotaxa.

Detrended Correspondence Analysis (DCA) [55] was used to produce a SK classification of 155 seagrass specimens using 21 morphometric characters, and a TEK classification using 22 characteristics from the ethno-classification surveys (i.e., morphology, ecology, experience, gestalt). The 21 morphometric characters used in the SK classification included: leaf arrangement, leaf complexity, leaf number, leaf width, leaf color, flower color, flower symmetry, ovary position, calyx number, calyx type, corolla number, corolla type, stamen number, stamen type, carpel number, carpel type, growth form, habit and other. Interset Pearson correlations were calculated between every characteristic and each of the first four axes in each ordination. Canonical ordination was used to detect groups of specimens and to estimate the contribution of each variable to the ordination. A Principal Component Analysis (PCA) [55] was used to identify the length of the ordination axis and the need for either a linear or unimodal ordination technique. Unimodal, indirect ordination Detrended Correspondence Analysis (DCA) was used to explore variation in species scores in this study. A cluster analysis was used to classify the specimens, as it is better in representing distances among similar specimens, whereas DCA is better in representing distances among groups of specimens [56]. Cluster analysis was performed with NTSYS [57]. A distance matrix was generated using an arithmetic average (UPGMA) clustering algorithm and standardized data based on average taxonomic distance subjected to the unweighted pair-group method.

## Results

### Identification of Seagrasses

**Figure 3:**
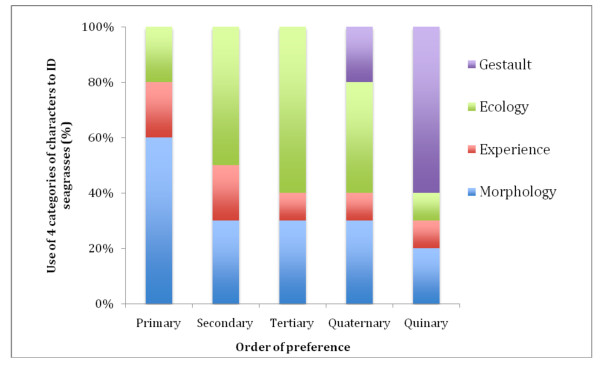
**Usage (%) of four ethno-classification categories ranked in order of preference (1^st^-5^th^ choice) to identify 155 specimens into 50 ethnotaxa**.

Although the ability of field taxonomists and local informants to identify species of seagrasses was high, the respective classifications of SK and TEK are not homogeneous. Taxonomists identified 10 species of seagrasses from 155 specimens with 88% (RF) accuracy among individuals. Species were identified through the use of floristic keys, and by comparing 21 vegetative and floral characters to known herbarium specimens. Local informants identified 50 seagrass ethnotaxa from the same 155 specimens with 95% (RF) accuracy among the informants. The primary characteristics used by informants to identify seagrasses fall into four categories: morphology, experience, ecology and gestalt. Morphological characters were used most often to recognize plants, and of these, vegetative features (78%) were more commonly used than floral features (22%). This may be because some of the seagrass species flower regularly, while others rarely have flowers (Figure 3). These vegetative and floral characteristics could be easily distinguished through visual inspection of the samples as they include several salient features that differ in shape, color and size. Ecological knowledge, such as knowledge of microhabitat and site conditions, is a key factor that is used by the locals to distinguish seagrasses (Figure 3). Personal experience with plants accounts for a considerable portion of the skills used by the locals to identify seagrasses (Figure 3). These experiences are hierarchical in usage and are comprised of olfaction (smell), palpation (touch), gustation (taste), auditory (sound) and visual cues, respectively. The last category called "gestalt" refers to the ability to recognize ethnotaxa as a whole without any reference to specific characteristics. Often the locals said that they could identify some ethnotaxa simply because they know them; elders and respected individuals had introduced them to specific ethnotaxa.

### Classification of Seagrasses

**Figure 4:**
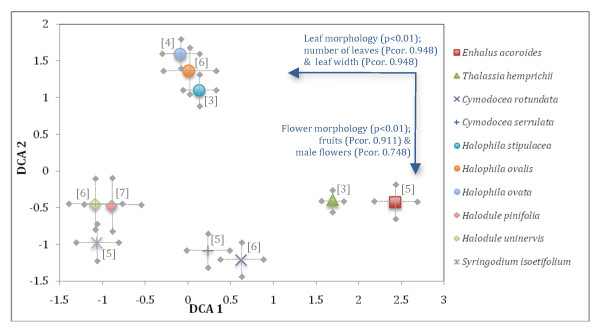
**Morphometric scatter plot of the first two axes from a Detrended Correspondence Analysis (DCA) for 155 specimens (classification of 10 seagrass species) constrained by 21 quantitative variables (taxonomic characters)**. Intraspecific variance is shown by error bars (2SD). Bi-plot arrows represent the strength of the correlations among explanatory variables and the first two DCA axes. Numbers of ethnotaxa are shown in parentheses for each species of seagrass indicating the fine-scale ethno-classification of intraspecific variation.

The morphometric classification indicates a clear separation of six groups from the 155 specimens that were analyzed. These six groups correspond with the six genera identified: *Enhalus, Halophila, Thalassia, Cymodocea, Halodule, and Syringodium *(Figure 4). Intraspecific variation prevents clear distinction among the ten species. Principal Components Analysis (PCA) provided a character gradient that was unimodal (4.5 SD), violating the assumption of a linear model. Consequently, a Detrended Correspondence Analysis (DCA) was used to classify the 155 specimens into groups representing 10 species. This is supported by eigenvalues that were moderate (0.192) for the first two DCA axes, and low for the third DCA axis (Table 1). Correlations among the explanatory variables and the first two gradients indicate the importance of vegetative and floral characters for the classification of 10 seagrasses (Figure 4). The first axis is significantly (p<0.01) correlated with the number of leaves (Pcor. 0.948) and leaf width (Pcor. 0.948). The second axis is significantly (p<0.01) correlated with fruit morphology (Pcor. 0.911) and the orientation of androecium (Pcor. 0.748).

**Table 1:** Summary of Detrended Correspondence Analysis (DCA) for the ordination of 155 specimens of seagrass classified using scientific (SK = 10 species) and traditional (TEK = 50 ethnotaxa).

**DCA Metrics**	**Classification**	**DCA Axis**			
		**1**	**2**	**3**	**4**
Eigenvalue	SK	0.075	0.052	0.007	0.004
	TEK	0.339	0.322	0.236	0.000
Cumulative % variance of species/ethnotaxa data explained	SK	53.4	90.8	95.4	98.0
	TEK	37.7	73.5	100.0	100.0

**Figure 5:**
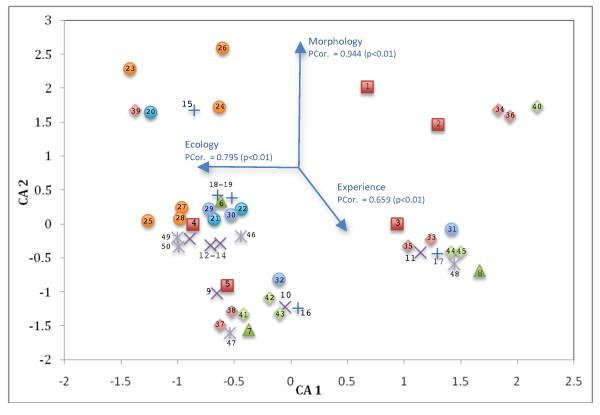
**Morphometric scatter plot of the first three axes from a Detrended Correspondence Analysis (DCA) for 155 specimens (classification of 50 seagrass ethnotaxa) constrained by 22 quantitative variables (ethno-taxonomic characters)**. Bi-plot arrows represent the strength of the correlations among explanatory variables and the first three DCA axes. Symbols for species are defined in the legend from Figure 3. Numbers within and beside symbols represents specific ethnotaxa of which the respective names can be found in Table 3.

The ethno-classification indicates considerable diversity at a finer scale than the SK classification. Intraspecific variation from the morphometric SK classification represented several ethnotaxa (Figure 4). Principal Components Analysis (PCA) identified a unimodal (5 SD) gradient in the ethno-classification analysis. Consequently, a Detrended Correspondence Analysis (DCA) was used to classify the 155 specimens into groups representing 50 ethnotaxa (Figure 5). This is supported by eigenvalues that were high (0.901) for the first three DCA axes and low for the fourth DCA axis (Table 1). Correlations among the explanatory variables and the first three gradients indicate the importance of morphology, ecology and experience for the classification of 50 seagrasses (Figure 5). The first axis is significantly (p<0.01) correlated with morphological characters (Pcor. 0.944), such as the type of leaf, color of the plant, or growth form. The second axis is significantly (p<0.01) correlated with ecological factors (Pcor. 0.795), such as microhabitat and site characteristics. The third DCA axis is significantly (p<0.01) correlated with experience factors (Pcor. 0.659), such as the taste or smell of the seagrass.

### Utility of seagrasses

There were many uses for seagrasses recorded in our surveys. Our research indicates a high level of consensus of TEK concerning utility of seagrasses within (Fic = 0.91) and among (Fic = 0.86) the local communities. The relative frequency (RF) of TEK from each individual ethnotaxa from the interviews was high (mean RF = 0.94 ± 0.04). The informant consensus of seagrass usage resulted in Fic ranging from 0.78 to 1.00 per utility category (Table 2). Many ethnotaxa (94%) have ecological utility as they are recognized as having a key role in the environment and serve as a measure of health of any coastal marine ecosystem (Table 3). The level of consensus among the informants concerning the ecology of seagrasses was considerably high (Fic = 0.78) given that almost all of the 50 ethnotaxa were associated with multiple ecological variables. Surprisingly, all of the ethnotaxa have medicinal utility with relatively high consensus (Fic = 0.80-1.00). Some of the medicinal uses listed included the maintenance of general health, and the treatment of a variety of ailments including heart problems, mental disorders, dermatological problems, various infections and gastrointestinal ailments (Table 3). Species that are found in high abundance such as *Thalassia hemprichii *(Ehrenb.) Asch*., Cymodocea serrulata*, (R.Br.) Asch. & Magnus and *Syringodium isoetifolium *(Asch.) Dandy are used as animal feed largely for goats and pigs serving as a viable enterprise for small-scale farmers along the coast. Relatively few species (35%) have aesthetic or spiritual utility among the coastal cultures. The consensus among informants was high (Fic = 0.97) regarding the use of seagrasses within coastal communities for animal feed, aesthetic or spiritual purposes.

**Table 2:** Ethnobotanical consensus index for traditional knowledge concerning the utility of 50 seagrass ethnotaxa.

**Utility category**	**Number of use reports (N_ur_)**	**Number of Exthnotaxa per use report (N_t_)**	**Informants' consensus index factor (F_ic_^a^)**
Heart	29	2	0.96
General health	40	5	0.89
Mental disorder	27	1	1.00
Dermatological	15	2	0.92
Infection	11	3	0.80
Gastrointestinal	32	2	0.96
Ecological	37	9	0.78
Animal feed	40	2	0.97
Spiritual	36	2	0.97
Aesthetic	36	2	0.97

^a^Fic = N_ur_-N^t^/(N_ur_-1), providing a value between 0 and 1, where high value indicates a high rate of informant consensus.

**Table 3:** Utility of 10 seagrass species, including habitat and etymology of 50 ethnotaxa.

**Ethnotaxa (Species & CBD Herbarium Accession Number**	**Habitats**	**Etymology & Main Utility**
*Cymodocea rotundata *Asch. & Schweinf.	Purely marine; shallow regions of tidal and subtidal zones; sandy and muddy substratum	**Id: **Rhizome creeping, branched, white to pale brown; shoots erect; sheaths pale white; stems less sweet but more salty; leaves straight and slightly falcate; lamina width narrow.
		**Etymology:$**(10) **Alai vaari: **Alai means waves, vaari means drifted or taking away; used together, Alai vaari means leaves floating along the wave.$(11) **Kadal korai: **Kadal means sea, korai means$sedge.$(12) **Kadal karumbu: **means sea sugar cane.$(13) **Kadal thazahi pasi: **Kadal means sea, thazahi means leafy, pasi refers to thalloid form.$(14) **Vellai kadal korrai pasi: **Vellai means white, kadal means sea, korrai means sedge, pasi means thalloid form of a seawater alga.$(9) **Vellai thazahi pasi**: Vellai means white, thazahi means *Pandanus *leaf type, pasi means thalloid form of a seawater alga.
		**Utility: **Branches and leaves used as feed for goats and pigs. Paste from leaves used to treat wounds. Collected biomass used as green manure for gardens.
*Cymodocea serrulata *(R.Br.) Asch. & Magnus	Purely marine; deep shallow regions of tidal and subtidal zones; sandy and muddy substratum	**Id: **Rhizomes creeping, branched; stems reddish brown to white; shoots long, more sweet and less salty, juicy; leaves falcate; lamina broad.
		**Etymology:$**(15) **Karumbu: **means sugar cane.$(16) **Karumbu pasi: **Karumbu means sugarcane thalloid, pasi means thalloid form of a seawater alga.$(17) **Kadal karumbu**: Kadal means sea, karumbu means sugarcane.$(18) **Peria korai pasi: **Peria means big, korai means sedge, pasi means thalloid.$(19)**Peria thazai pasi: **Peria means big, thazai means *Pandanus *leaf type, pasi means thalloid.
		**Utility: **Branches and leaves used as feed for goats and pigs. Paste from leaves used to treat wounds. Collected biomass used as green manure for gardens.
*Enhalus acoroides *(L.f.) Royle	Pure marine; shallow water; sandy to fine mud substratum	**Id: **Leaves long, dark green, ribbon-like; rhizome very thick; roots stout; female inflorescence on long peduncles, single flowered like a lily.
		**Etymology:$**(1) **Olai pasi**: Olai means palm leaf, pasi means thalloid form of a seawater alga.$(2) **Vaata alai: **Vaataa means running toward the wave, alai means waves; together name means the direction the leaves float along the waves.$(3) **Alai vaari**: Alai means waves, vaari means floating away; together name means leaves that float along the wave.$(4) **Kadal vaari: **Kadal means sea, vaari means floating away; together name means leaves swaying in the wave.$(5) **Thittu pasi**: thittu means patches, pasi means thalloid form of a seawater alga.
		**Utility: **Rhizome and root juices consumed raw to treat seasickness. Rhizome (peeled of skin) consumed fresh with cup of seawater to treat heart conditions and low blood pressure. Rhizomes consumed fresh to ease indigestion and hangover. Paste of fresh leaves used to treat many kinds of skin diseases. Seeds, which taste like almonds, are eaten by people or fed to goats and sheep.
*Halodule pinifolia *(Miki) Hartog	Tidal and subtidal zones; fine sandy and muddy bottoms; mangrove creeks and swamps	**Id: **Rhizomes creeping, white to pale brown; shoots long, erect; leave flat, long.
		**Etymology:$**(34) **Nedung korai**: Nedung means long, korai refers to sedge.$(35) **Neettu korai: **Neettu means long, korai means sedge.$(36) **Korai: **Korai means sedge.$(37) **Kadal korai**: Kadal means sea, korai means sedge.$(38) **Kadal korai pasi**: Kadal means sea, korai means sedge, pasi means thalloid form of a seawater alga.$(33) **Arugampul pasi**: Arugampul refers to the terrestrial plant called *Cynodon dacylon*, which is traditionally called Arugampul; pasi means thalloid form of a seawater alga.$(39) **Peria eekku thazhai**: Peria means long/big, eekku means sharp tiny stick (like tooth pick), thazhai refers to terrestrial plant of the *Pandanus *genus.
		**Utility: **Stems are washed and used as goat feed.
*Halodule uninervis *(Forssk.) Boiss.	Purely marine; deeper tidal and subtidal zones in muddy bottoms	**Id: **Rhizomes creeping, white to pale brown; shoots short, erect; leave flat, narrow, short;
		**Etymology:$**(41) **Kothu korai: **Kothu means forms in patches, korai means sedge.$(42) **Kattai korai: **Kattai refers to stout-strong stem, korai means sedge.$(43) **Pullu korai**: Pullu means grass, korai means grassy sedge.$(44) **Korai: **Korai means sedge.$(45) **Sinna eekku thazahi: **Sinna means small, eekku refers to a sharp tiny stick, thazhai refers to terrestrial plant of the *Pandanus *genus.$(40) **Panjipul pasi: **Panjipul means the whole plant is soft to the touch, and tiny; pasi means thalloid form or seaweed.
		**Utility: **Stems are washed and used as goat feed.
*Halophila ovalis *(R.Br.) Hook.f.	Tidal and subtidal zones; coral debris and muddy substratum	**Id: **Rhizome creeping, branched, slender, semi-transparent, fleshy, brittle; lamina oblong-elliptic, pale to dark green.
		**Etymology:$**(25) **Kadal sedi pasi: **Kadal means sea, sedi means herb, pasi means thalloid form or seaweed; together the name means flat seaweed.$(26) **Kouthu pasi: **Kouthu means bunch, pasi means thalloid form or seaweed.$(23) **Murungai pasi: **Murungai refers to *Moringa *plant, pasi means thalloid form or seaweed$(28) **Sedi pasi: **Sedi means herb, pasi means thalloid form or seaweed.$(24) **Seathu pasi: **Seathu means mud, pasi means thalloid form or seaweed.$(27) **Kadal pasi: **Kadal means sea, pasi means thalloid form or seaweed; together name means seaweed.
		**Utility: **A handful of leaves is toasted with three drops of sesame oil and consumed for three days to treat iron deficiency. A leaf paste is mixed with turmeric and applied to cure various skin ailments, including burns and boils.
*Halophila gaudichaudii *J.Kuo (syn. ***Halophila ovata *Gaud.)**	Low tide, wave-less open sea; coarse sandy and soft muddy bottoms	**Id: **Rhizome slender, terete, fleshy, unbranched, semi-transparent; leaves oblong - elliptic, linear-oblong.
		**Etymology:$**(31) **Sedi pasi: **Sedi means herb, pasi means thalloid form or seaweed; together the name means herb type pasi.$(32) **Elai pasi: **Elai means leaf, pasi means thalloid form or seaweed; name means leaf-like structure found in sea.$(29) **Poduthali**: Poduthali refers to *Phyla nodiflora*, the leaf of which is is brittle.$(30) **Pottal pasi: **Pottal refers to the plain sandy substratum where this plant is found, pasi means thalloid form or seaweed.
		**Utility: **A handful of leaves is toasted with three drops of sesame oil and consumed for three days to treat iron deficiency. A leaf paste is mixed with turmeric and applied to cure various skin ailments, including burns and boils.
*Halophila stipulacea *(Forssk.) Asch.		**Id: **Herbaceous and creeping; runner slender, transparent, brittle; lamina lanceolate; pedicle short.
		**Etymology:$Minni pasi**: Minnal means lightning, pasi means thalloid form or seaweed; name refers to the reflective light and color of water.$**Kadal pasi **Kadal means sea, pasi means thalloid form or seaweed; a thalloid looking seaweed*.$***Sedi pasi: **Sedi means terrestrial seaweed, pasi means thalloid form or seaweed.
*Syringodium isoetifolium *(Asch.) Dandy	Purely marine; occur in deeper zones on coral flats; sandy to muddy bottoms	**Id: **Rhizomes creeping, slender, semi-fleshy, white to pale yellow; shoots long; leaves long, lamina terete, succulent/fleshy and thick.
		**Etymology:$**(46) **Neer pasi: **Neer refers to the watery substance that comes out of the succulent leaves when squeezed, pasi means thalloid or seaweed type.$(47) **Oosi korai: **Needle type sedge.$(48) **Neer korai**: Neer refers to the watery substance, korai means sedge; a watery/fleshy sedge.$(49) **Korai pasi**: Korai means sedge, pasi means thalloid or seaweed type.$(50) **Nool pasi**: Thread like thalloid form.
		**Utility: **Fresh leaf juice consumed to ease acid reflux. Stems fed to goats. Biomass used as green manure for garden.
*Thalassia hemprichii *(Ehrenb. ex Solms) Asch.	Purely marine; tidal and subtidal zones; black muddy, and loose sandy soils	**Id: **Rhizomes creeping, branched, brittle; leaves falcate; shoots brownish-black, internodes short, many.
		**Etymology:$**(6) **Kattai korai pasi: **Kattai means woody and refers to the plant's stout rhizomes, korai means sedge, pasi means thalloid or seaweed type.$(7) **Korai pasi: **Korai means sedge, pasi means thalloid or seaweed type.$(8) **Kadal korai: **Kadal means sea, korai means sedge.
		**Utility: **Dried rhizome powder consumed to treat mental disorders. The same powder mixed with coconut oil and applied on wounds. Biomass used as green manure for gardens.

(Herbarium accession voucher numbers are provided for the Center for Biocultural Diversity in Chennai, India, and the ARK collection at the Biodiversity Institute of Ontario, University of Guelph).

## Discussion

### Classification of Seagrasses

Our interpretation of diversity is entirely dependent on the classification lens with which we view the landscape. The coastal landscape of Tamil Nadu, India is abundant with seagrasses. A scientific classification lens provides resolution at a scale that identifies 12 species, 10 of which are important to local cultures. Two tiny (<1.5cm) species (*Halophila decipiens *and *Halophila beccarii*) are restricted to specific habitats and are recognized, but not used by the local cultures. These cultures appear to use a finer scaled lens for classification than SK, based on a system of traditional knowledge that has been in place for centuries, if not longer. This traditional classification interprets intraspecific variation revealing 50 ethnotaxa that have considerable utility among the local villages along the coast. This was consistent both in the field and when specimens were displayed within local villages. This is not the first account where TEK classification systems record diversity at a finer-scale than SK systems. In a review of indigenous biological classification systems [50,58], we found a handful of studies where indigenous informants recorded more taxa in a particular area than taxonomists using a SK classification system [59-61]. However, none of these studies allowed a direct comparison within an experimental design in the same area. Our earlier research incorporated such a research design with the Irulas in a remote area of Tamil Nadu and discovered that for surveys of a diverse group of plants in exactly the same area, the Irula TEK classification system was more robust than the SK classification [50]. Our seagrass study corroborates this earlier evidence [50,58], which defines a multi-mechanistic model for classification systems based on traditional knowledge. The direct comparison of SK and TEK classifications of 155 seagrass specimens was not congruent. This is because the TEK classification system considers other classification variables such as ecology and sensory perception (e.g., smell, taste), differentiating seagrasses at a very fine-scale. The TEK classification does not align itself to the SK classification; ethnotaxa are not clustered within a species concept, such as subspecies or varieties. Instead, the ethnotaxa data cluster with respect to morphology, ecology, experience and gestalt categories. This illustrates that local coastal cultures have a classification based on both salient and functional characters. This evidence of classification using the latter functional characters reflects a natural interpretation of seagrass diversity based on how these species respond to ecological gradients. Perhaps TEK classification is more aligned with evolutionary principles as these cultures have had the opportunity to observe how species respond to different environments. This natural interpretation of the variation in taxa is critical for those cultures that utilize seagrasses on a day-to-day basis. The need to classify is underpinned by the utility of the various seagrass ethnotaxa, which has been entrenched within these coastal cultures for centuries.

### Variation in Seagrass Morphology

The TEK interpretation of morphological variation among seagrasses is very detailed. The morphological characteristics used within the TEK classification are similar to that of the SK classification, including plant size (e.g., height, leaf width) or features such as rhizomes (e.g., size, shape and structure), seeds (e.g., colour, shape and lustre) or stems (e.g., diameter, colour). For example, some fishermen recognize Minnipassi (*Halophila stipulacea*) based on color alone. All of the species within the *Halophila *complex look morphologically similar when viewed through a SK lens; using characters found in the SK classification system. However, TEK morphological characters can differentiate much more variation within the *Halophila *complex, including the ethnotaxa Minnipassi, which is distinguished by the color of its leaves in its natural habitat; the leaves are a variegated pinkish brown color and float on water with a distinctive sparkle and radiant colour (Minnal means sparkling/lighting). *Halophila ovata *and *Halophila ovalis *look very similar macroscopically, but fishermen can recognize these species by comparing them to leaves from the terrestrial plants *Moringa oleifera *and *Phyla nodiflora*. Kouthupasi (*H. ovalis*) leaves are similar in size and shape to *M. oleifera*, and Poduthali passi (*H. ovata*) has leaves that are similar to *Phyla nodiflora*. The *M. oleifera *leaves are popular among fisherman for use as an ingredient in a vegetarian soup.

The *Halodule spp*. complex is also difficult for SK to differentiate. Local people call this group of plants 'Eekku passi'. The name 'Eekku' could be used as a substitute for *Halodule*, and it refers to the tip of the leaves, which can be oblique, dentate, serrate and minute spiny. The ethnotaxa 'Sinna eekku passi' (*Halodule uninervis*) refers to the slightly shorter leaf of this species; 'sinna' means 'the leaf is short'. The ethnotaxa 'Peria eekku passi' (*Halodule pinnifolia*) has longer leaves; 'peria' means 'the leaf is longer'. Another ethnotaxa called 'Panjipull passi' (*H. uninvervis*) is distinguished by its soft texture. Local people identify the ethnotaxa 'Arugampul passi' (*H. pinnifolia*) by comparing it to a similar looking terrestrial plant of a different genus (*Cynodon dacylon*), which locals call 'Arugampul'. Other seagrasses in genera such as *Thalassia *and *Cymodocea *look similar to sedges, and as a general name, the TEK classification regards this group as 'Kattai korai'. Kattai refers to the genus *Thalassia*, which has a thick and stout rhizome, whereas korai refers to the genus *Cymodocea*. The fishermen easily distinguish all species in these two genera.

Many traditional cultures around the world identify and classify plants using sensory perception, namely sight, touch, taste, smell and sound. In our study, informants identified many seagrasses using experiential characters. For example, the ethnotaxa 'Neer pasi' (*Syringodium isoetifolium*) is identified by the texture of the watery substance that comes out of its succulent leaves when squeezed; 'Neer pasi' means 'watery substance'. Similarly, the two species of the *Cymodocea spp*. complex, *Cymodocea serrulata *and *Cymodocea rotundata*, look more or less similar to scientists macroscopically. However, as perceived by local people, *C. serrulata *(Karumbu pasi; Karumbu means sugar cane) is sweeter than *C. rotundata *(Vellai thazhai pasi). 'Vellai thazhai pasi' is sometimes misidentified as 'Vellai karumbu pasi', which is sweeter than 'Vellai thazhai pasi', and saltier than 'Karumbu pasi'.

### Variation in Seagrass Ecology

TEK uses fine-scaled variation in seagrass habitat and ecology. In this study, many people in the fishing communities recognize sea grasses by their associated habitat. For example, local people see the black patches over water bodies in shallow regions during high or medium tides as an indication of the presence of submerged seagrass beds. Locally, these patchy areas are called the 'Saethupasi', and they are an easy way for people to locate seagrass. 'Saethupasi' zones can be sandy or muddy, stretching as an extensive scattered pattern half a kilometer out to sea, and within these zones, many different seagrass taxa inhabit specific ecological niches. These niche relationships are recognized and used by locals to identify the different ethnotaxa. As an example, 'Pottal pasi' (*H. ovata*) is found in clear water zones where there is a sandy substratum called 'Pottal', which means 'useless'. The term "pottal" is used among the fishermen to describe clear water areas where fish populations are low (presumably because of low nutrient content); hence, local fishermen view these areas as 'useless' for fishing. The ethnotaxa 'Saethu pasi' (*H. ovalis*) is found in dark water zones that have a muddy substratum. Fishermen find these muddy or 'saethu' zones very interesting because they provide habitat for numerous crabs, shrimps and fish. Although it is difficult for a trained taxonomist to distinguish *H. ovata *('Pottal pasi') from *H. ovalis *('Saethu pasi') without using macroscopic characters (e.g., seed number and cross venation pattern), local people can easily distinguish the two species based on the different habitats they grow in.

### Seagrass Ethnomedicine and Nutrition

**Figure 6:**
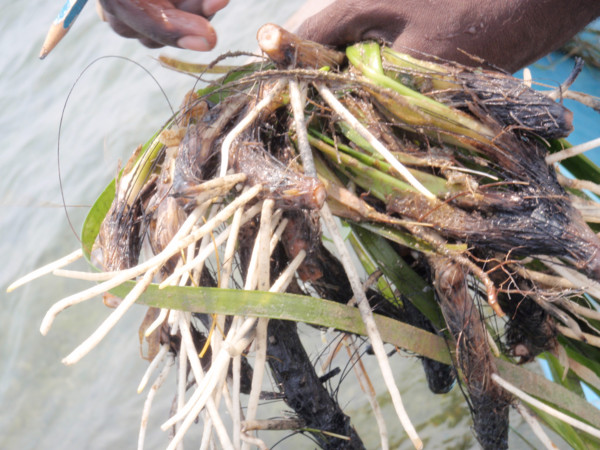
**Fresh rhizomes of Olai pasi (*Enhalus acoroides*), which is used to treat heart conditions**.

There is very little information in the literature concerning the traditional use of seagrass as ethnomedicine. In research by de la Torre-Castro and Rönnbäck [21], seagrasses are reported as treatment for a number of ailments in a rural economy in Zanzibar, including stings, muscle pain, wounds, stomach problems, fever, malaria and coughs, among others. Another study, Felger and Moser [14], describes the use of seagrass by the Seri Indians in Mexico as treatment for diarrhea. However, none of these claims are supported by survey data, quantitative analyses, consensus or any other kind of analysis typical for ethnobiology research. Our study provides the first detailed account on the traditional knowledge of seagrasses used as ethnomedicine. We provide detailed records of 12 ailments (wounds, sea sickness, heart disease, low blood pressure, indigestion, hangover, skin disease, iron deficiency, burns, boils, acid reflux and mental disorders) for which 50 ethnotaxa serve as treatment. For serious medical conditions such as heart disease and low blood pressure, people peel off the skin and eat the fresh rhizome of Olai pasi (*Enhalus acoroides*) with a cup of sea-water (Figure 6). Locals also use seagrasses to treat common ailments such as dandruff; the fresh leaves of 'Poduthali pasi' (*H. ovata*) are ground in to paste and applied onto the scalp for a week. It is interesting to note that another terrestrial ethnotaxa called 'Poduthali' (*Phyla nodiflora*) is also used to control dandruff, but this plant belongs to the Verbenaceae family and is not a seagrass. The leaves of both *P. nodiflora *and *H. ovata *are brittle. For mental illnesses, people use the dried rhizome powder of ethnotaxa 'Kadal korai' (*Thalassia hemprichii*) as a treatment. For iron deficiencies, a handful of 'Elai pasi' (*H. ovata*) leaves is mixed with sesame oil and consumed daily for three days. Various skin diseases, burns and boils are treated with the leaves of 'Murungai pasi' (*H. ovalis*) and a paste made from turmeric. Many of the seagrasses are used for general good health.

**Figure 7:**
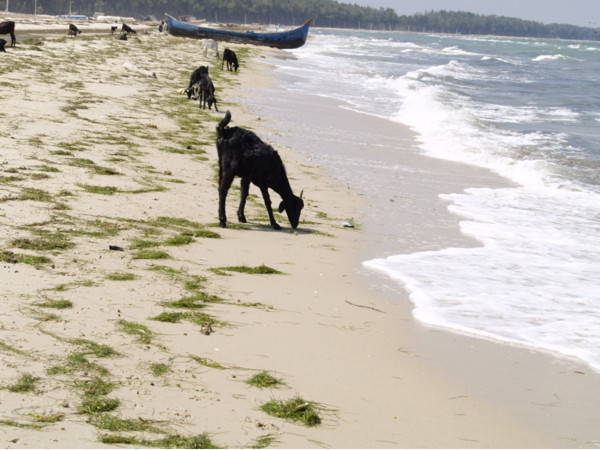
**Goats grazing on seagrasses along the shoreline pasture**.

Seagrasses have nutritional value for people and livestock. Some ethnic groups in the Philippines [62] and in Mexico [13,14] have traditionally used the seeds of seagrasses as food. Our study revealed similar findings. Eaten fresh, 'Vattalai' seeds (*Enhalus acoroides*) taste sweet and crunchy like groundnuts or almonds and are commonly eaten by locals in many of the coastal villages in this study, including Pamban, Munaikadu, Ariaman, Devipattinam, Thondi, Pasipattinam, Namputhazai, Thirupazhkudi, Mimisal and Kattumavadi. The local fishermen often eat Vattalai seeds while out fishing, saying it increases their energy. They also claim that eating the seeds increases libido, a finding also discussed by Montaño et al. [62]. *Enhalus acoroides *seeds are high in starch, with energy values similar to terrestrial plant flours and starches, and when cooked the seeds taste starchy like sweet potato [62]. In this study, villagers used these seeds as feed to bulk up livestock such as goats, sheep, pigs and cattle (Figure 7). During the dry season, livestock are herded onto the shore where they left to graze on seagrass, consuming large quantities of seeds. However, we were told that the animals would get sick and die if they feed on seagrass during the rainy season; thus, animals are kept away from the shore during this time of year. We were also told that the sick animals would search for and consume 'Neer pasi' (*Syringodium isoetifolium*), which could apparently cure their illness.

**Figure 8:**
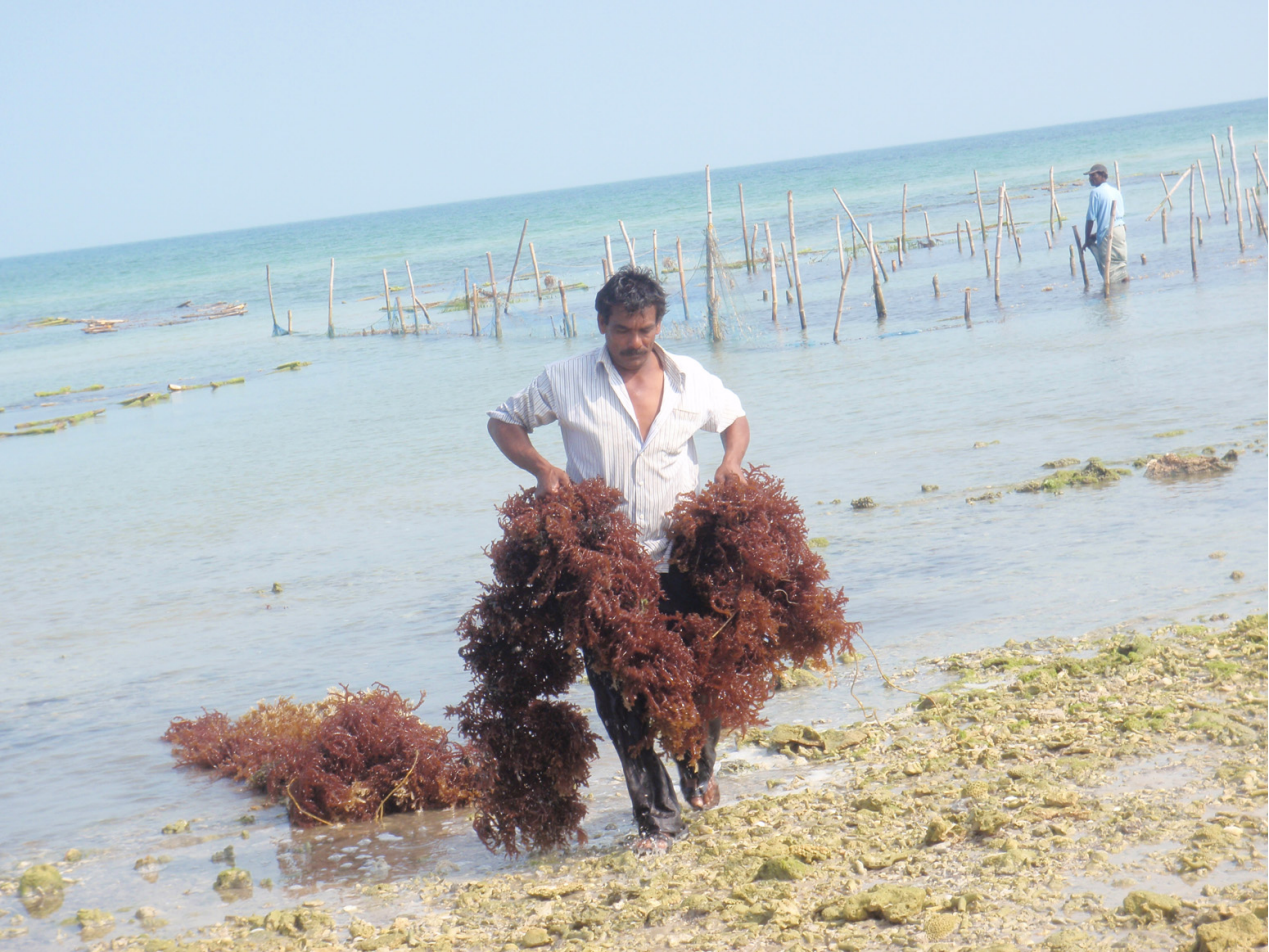
**Fishermen bringing seaweeds from the sea for sorting**.

**Figure 9:**
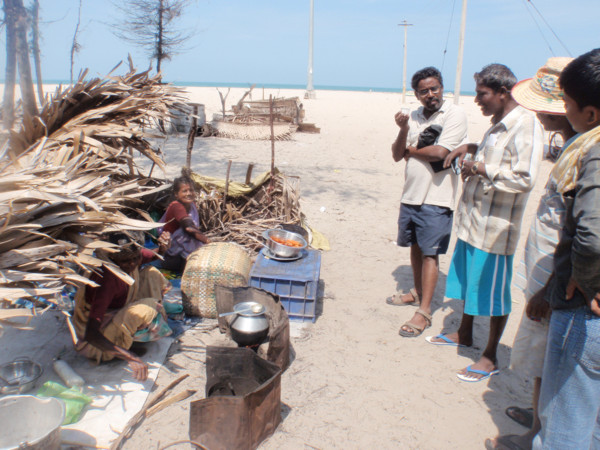
**Shoreline depot where local people trade or buy seagrass by-products (Vatala payasam refers to 'seagrass pudding')**.

This research found that the leaves of seagrasses were also used locally for a number of other practical functions. First, seagrass leaves of *Cymodocea serrulata *and *Thalassia hemprichii *were used by local fishing market as insulation during the hot summer to keep stored ice and fish cool. Second, the biomass of seagrasses that accumulates on the shore is considerable and the locals use this as manure in coconut and tobacco plantations. Wyllie-Echevarria and Cox [11] also discuss the use of seagrasses as insulation and green manure. For the coastal community of Yarmouth County, Nova Scotia, the authors provide a detailed account of the traditional and commercial importance of *Zostera marina *gathering during the early to mid 1900s. In our study, the importance of seagrasses to local coastal economies in the state of Tamil Nadu is apparent by the engagement of the community in collecting and sorting seagrasses (Figure 8). In fact, trading depots are established along the coast where people trade or buy local produce including seagrasses (Figure 9).

### Seagrass Ecosystem Biodiversity

The Coromandel Coast on the Gulf of Mannar is the first marine biosphere reserve in South Asia, and harbors all 12 species of seagrasses found in India. This biodiverse ecosystem is supported by a variety of substratum. The islands of this gulf are surrounded in large part by coral rubble, coral reefs, sandstone, sand and mud, providing ideal habitat for the luxurious growth of the many seagrasses including species of *Cymodocea, Enhalus, Halodule, Thalassia *and *Syringodium*. The predominant species are *Cymodocea rotundata, C. serrulata, Halodule pinifolia, Halophila ovalis, Syringodium isoetifolium *and *Thalassia hemprichii*. These seagrasses provide critical habitat for diverse marine fauna such as cuttlefish, dugongs, sea horses, eels, rays and scorpion fish, sea cucumbers and sea snakes, among others. Seagrass species in the *Enhalus *genus serve as habitat for sea snakes, and can thus prove perilous to the unwary as the seagrass leaves resemble the snakes themselves, providing good camouflage. The locals told us that sea snakes gather on the leaves of these seagrasses every year at a certain time to engage in pseudo-copulation, which we observed in our research sites near Kattumavdi, Thirupazhkudi and Thondi. Some of the less predominant seagrasses along the Coromandel Coast include *Halophila beccarii, Halodule uninervis *and *Halophila ovata*. These species are found in specific, sometimes sheltered habitats that are known to local informants.

Palk Bay is a shallow sheltered bay with extraordinary seagrass diversity. Here the substratum is uniformly loose, black and muddy, with some sand, and locals explained that there are two distinct habitat zones in the bay that are recognized by the types of seagrasses they host. The first of these habitats is a shallow marginal zone dominated by *Halodule spp*. and *Halophila spp.*, while a second, deeper zone is located approximately a half kilometer from shore and hosts species of *Cymodocea, Enhalus *and *Syringodium*. The most common seagrasses in Palk Bay are *Cymodocea serrulata, Halodule pinifolia, Halophila ovalis *and *Syringodium isoetifolium*. Uncommon species include *Halodule uninervis, Halophila ovata *and *H. stipulacea*. *Enhalus acoroides *is located in isolated patches and is restricted to Kattumavadi, Mimisal, Thirupazhkudi, Namputhazai, Passipattinum, Thondi, Devipattinam and Ariaman. Local fishermen are concerned that an increased frequency of violent tidal action is responsible for the loss of seagrass habitat. They say that it is getting difficult to collect certain seagrass ethnotaxa and that good fishing sites are increasingly harder to find. These changes have important consequences for coastal communities that have been dependent on seagrass ecosystems for centuries.

Mangrove creeks and river estuaries are important traditional seagrass collection sites. Although seagrass diversity is low compared to that found in Gulf of Mannar and Palk Bay, informants impressed upon us how vital these ecosystems are for collecting large quantities of specific ethnotaxa. Estuaries near the mouth of rivers harbor extensive beds of *Halodule spp*. and *Halophila spp*., while some of the other seagrass species found (e.g., *Cymodocea rotundata, C. serrulata, Enhalus acoroides, Syringodium isoetifolium *and *Thalassia hemprichii*) drift in after storms. The substrata of these estuaries are usually clayey to black muddy with fine to coarse sand. There is concern among the local communities that the depletion of seagrasses in these backwaters might be due to an unstable sandy substratum associated with certain anthropogenic disturbances on the rivers, such as dams, irrigation for large-scale farming and building developments.

### Littoral Drift and Seagrasses

An important issue concerning the conservation of seagrasses lies in the erosion of spits, shoals, and the coastline in general. While much of this erosion is due to concentrated wave energy, there is also a growing loss of natural sediment aggradation by rivers due primarily to upstream damming and irrigation barrages [63]. This lack of deposition results in a net loss of seagrass habitat and the nutrients that would otherwise be deposited with the sediment, and produces a cascade of biological and ecological problems. Seagrasses are an important sediment stabilizer as they provide a vital buffer against wave action [2], and with wave action left unimpeded, the degradation and erosion of the shoreline is inevitable. Seagrasses are also important in recycling nutrients and filtering contaminants in the transition zone between land and sea. They provide innumerable resources for thousands of plant and animal species that rely on land-sea transition zones for protection, breeding, sustenance and habitat, and are thus of vital importance to the overall health of ocean life [2]. Critical action is therefore needed for the conservation of seagrass ecosystems, and such action must consider the assemblage of both scientific and traditional knowledge.

### Seagrass Ecosystem Conservation

The importance of seagrass meadows to estuarine and coastal marine ecosystems is generally underestimated. Seagrasses have a high growth rate, produce abundant biomass, and they support major food chains including direct grazers and animals that feed on their epiphytic associates, as well as the decomposer communities that are sustained by seagrass detritus [64]. Seagrasses also contribute to an active sulfur cycle through decomposition, increase sedimentation of organic and inorganic materials by the buffering action of leaves, and reduce surface erosion by the sediment binding action of roots, thereby preserving the microbial flora of the sediment and the sediment-water interface. Following from these diverse roles, seagrass meadows require special management and conservation strategies. Research is needed to understand the abiotic factors (e.g., light, temperature, salinity, nutrient cycling, substratum, ocean currents and tidal rhythms), as well as the natural and anthropogenic disturbances that shape seagrass community composition. Local communities are often the first to notice environmental change due to these forces and factors. As an example, local people interviewed in this study reported decreases in productive fishing habitat, and a recent increase in the scale and frequency of disturbances, particularly in riverine systems that feed into estuaries. These disturbances include natural phenomena such as floods and wave action, as well as the following large-scale anthropogenic disturbances: forestry; irrigation; dams; channeling and stream diversion; sewage treatment discharge; and soil erosion from inland development [27]. However, anthropogenic disturbance also occurs at the local level. In this study, some of the more serious concerns with regard to seagrass habitat degradation at the community level were discharge of household wastes and use of mechanized boats. Understanding this full assortment of factors that drive and shape seagrass communities is essential for developing integrated conservation strategies that support the complex seagrass food web and their associated faunal relationships.

Although conservation measures for seagrass ecosystems have been established in marine biodiversity hotspots around the world, such as in North America and Australia, there are no adaptive management strategies, public education plans or conservation measures in place for seagrass ecosystems in India. As a first step towards a management strategy for seagrass ecosystems, it is already well understood that the success of any integrated science-based strategies require effective conservation management in the field at the local level [28]. However, as a further step, an adaptive management strategy would facilitate the assemblage of multiple knowledge systems (SK and TEK), public education and the initiation of conservation policy in an iterative process that would evolve as we learn how to preserve these delicate ecosystems. This could start by bringing local communities, scientists, resource managers and government officials together in symposia to design an action plan for seagrass conservation. Such a strategy would be in accordance with Agenda 21, Chapter 17, of the 1992 Earth Summit (Rio de Janeiro, Brazil), which states that government agencies charged with coastal zone protection must integrate TEK and socio-cultural values with management agendas [65].

The importance of seagrass ecosystems to traditional cultures has been neglected and deserves stronger recognition and respect. Historically, this resource has sustained coastal livelihoods for centuries based on their agricultural (fishing and farming) and medicinal utility. In this study, the locals shared with us their belief that a healthy ecosystem supports healthy livelihoods. Elders told us that that the sustenance of life in their communities is dependent upon stable seagrass habitat, and that it is for this reason that traditionally they have moved their communities in search of healthy seagrass ecosystems. Seagrasses are fundamental components of healthy marine ecosystems and the local livelihoods that rely on them.

## Consent

Written informed consent was obtained for publication of accompanying images. A copy of the written consent is available for review by the Editor-in-Chief of this journal.
